# Continuing professional development

**DOI:** 10.1002/jmrs.419

**Published:** 2020-09-07

Maximise your CPD by reading the following two selected articles which appear in this issue and answer the five questions. Please remember to self‐claim your CPD and retain your supporting evidence. Answers will be available via the QR code and online at https://www.asmirt.org/news-and-publications/jmrs, as well as published in the subsequent JMRS issue.

## Medical Imaging – Original Article

### Finding ways to support radiographers as teachers

Thompson A, Taylor D. (2020)

*J Med Radiat Sci*. https://doi.org/10.1002/jmrs.399

1. Radiographers in this study mostly learned to supervise by:
  - Undertaking a formal course/s in supervision
  - Continuing professional development opportunities
  - Trial and error, experience of being a learner, and observing the teaching practice of others
  - Attending supervision workshops
2. In relation to *facilitating students’ learning,* radiographers in this study demonstrated a:
  - High level of confidence (> 75%) *in stimulating students to apply research to clinical learning situations*
  - High level of confidence (> 75%) *in teaching and role‐modelling professional skills*
  - High level of confidence (> 75%) *in applying teaching techniques/strategies to facilitate student learning*
  - High level of confidence (> 75%) *in identifying strategies for students to grow and change*
3. The results of this study show that there was:
  - A significant difference in mean confidence ratings for *years of practice* and *location* (rural or metropolitan)
  - No difference in mean confidence ratings for *years of practice* and *location* (rural or metropolitan)
  - A significant difference in mean confidence ratings for *years of practice* but not *location*
  - A significant difference in mean confidence ratings for *location* but not *years of practice*
4. In this study, radiographer participants identified they would like additional support for their teaching role in relation to:
  - Technical ability
  - Communication skills
  - Practice knowledge
  - Strategies to manage struggling students
5. The findings of this study recommend that teaching‐focussed professional development for radiographers includes:
  - Workshops
  - Mentoring relationships formed between experienced and less experienced radiographers who teach students
  - Online initiatives (a resource/s to support and guide radiographers in their teaching role)
  - All of the above

## Recommended further reading

1. Cooper E, Neep MJ, Eastgate P. Communicating traumatic pathology to ensure shared understanding: is there a recipe for the perfect preliminary image evaluation? *J Med Radiat Sci* 2020; **67**: 143‐50. https://doi.org/10.1002/jmrs.375
2. McInerney J, Druva R. Clinical educators’ attitudes towards the use of technology in the clinical teaching environment. A mixed method study. *J Med Radiat Sci* 2019; **66**: 72‐80. https://doi.org/10.1002/jmrs.335
3. Sapkaroski D, Baird M, McInerney J, Dimmock MR. The implementation of a haptic feedback virtual reality simulation clinic with dynamic patient interaction and communication for medical imaging students. *J Med Radiat Sci* 2018; **65**: 218‐25. https://doi.org/10.1002/jmrs.288

## Radiation Therapy – Original Article

### New Zealand radiation therapists’ perceptions of peer group supervision as a tool to reduce burnout symptoms in the clinical setting

Dungey G, Neser H, Sim D. (2020)

*J Med Radiat Sci*. https://doi.org/10.1002/jmrs.398

1. According to the Maslach Burnout Inventory (MBI) which of the following is not a stage of burnout:
  - Emotional exhaustion
  - Depression
  - Depersonalisation
  - Decreased sense of personal accomplishment
2. Which of the following is **not** true of Peer Group Supervision (PGS)?
  - PGS allows staff to focus on interpersonal skills
  - PGS meetings should have 4‐6 members
  - PGS is led by a professional supervisor
  - PGS allows staff to manage challenging clinical situations
3. The factor analysis from this study found which of the following three factors?
  - Confidence in practice, team support, and group safety
  - Purpose, process, and impact
  - Team support, group safety, and purpose
  - Confidence in practice, team support, and group membership
4. Problems with PGS processes were largely due to?
  - Membership and staff issues
  - Group structure and time issues
  - Leadership issues
  - Membership and group structure issues
5. Which group of radiation therapists thought PGS may be useful in reducing burnout because they perceive it to improve patient care?
  - 0‐5 years of experience
  - 6–10 years of experience
  - 11–15 years of experience
  - 21–25 years of experience

## Recommended further reading

1. Jasperse M, Herst P, Dungey G. Evaluating stress, burnout and job satisfaction in New Zealand radiation oncology departments. *Eur J Cancer Care* 2014; **23**: 82–88. https://doi.org/10.1111/ecc.12098
2. Kuipers P, Pager S, Bell K, Hall F, Kendall M. Do structured arrangements for multidisciplinary peer group supervision make a difference for allied health professional outcomes? *J Multidiscip Healthc* 2013; **10**: 391–97. https://doi.org/10.2147/JMDH.S51339
3. Dawber C, O’Brien T. A Longitudinal, comparative evaluation of reflective practice groups for nurses working in intensive care and oncology. *J Nurs Care* 2014; **3**: 1–8. https://doi.org/10.4172/2167-1168.1000138

## Answers to Questions Published in Previous Issue

Please see JMRS Volume 67, Issue 2, June 2020 for the articles and CPD questions at https://doi.org/10.1002/jmrs.401.

Summerfield J, Leong A. Management of radiation therapy‐induced vaginal adhesions and stenosis: a New Zealand survey of current practice. *J Med Radiat Sci* 2020; **67**: 128–133. https://doi.org/10.1002/jmrs.386


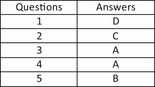


O’Keefe JR, Wilkinson JM, Spuur KM. Current practice in mammographic imaging of the augmented breast in Australia. *J Med Radiat Sci* 2020; **67**: 102–10. https://doi.org/10.1002/jmrs.374


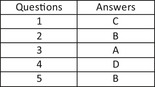


## Answers to this Issue


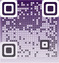


Scan this QR code to find the answers, or visit https://www.asmirt.org/news-and-publications/jmrs

